# A content analysis of alcohol content in UK television

**DOI:** 10.1093/pubmed/fdy142

**Published:** 2018-10-14

**Authors:** Alexander B Barker, Kathy Whittamore, John Britton, Rachael L Murray, Jo Cranwell

**Affiliations:** 1 UK Centre for Tobacco and Alcohol Studies, Division of Epidemiology and Public Health, University of Nottingham, Clinical Sciences Building, City Hospital, Nottingham, UK; 2 Tobacco Control Research Group, Department for Health, University of Bath, 1 W 5.124, Claverton Down, Bath, UK

**Keywords:** alcohol, content analysis, public health, television

## Abstract

**Background:**

Exposure to audio-visual alcohol content in media is associated with subsequent alcohol use in young people, but the extent of exposure contained in UK free-to-air prime-time television has not been explored since 2010. We report an analysis of alcohol content in a sample of UK free-to-air prime-time television broadcasts in 2015 and compare this with a similar analysis from 2010.

**Methods:**

Content analysis of all programmes and advertisement/trailer breaks broadcast on the five national UK free-to-air channels in the UK between 6 and 10 pm during three separate weeks in September, October and November 2015.

**Results:**

Alcohol content occurred in over 50% of all programmes broadcast and almost 50% of all advert/trailer periods between programmes. The majority of alcohol content occurred before the 9 pm watershed. Branding occurred in 3% of coded intervals and involved 122 brands, though three brands (*Heineken, Corona* and *Fosters*) accounted for almost half of all brand appearances.

**Conclusion:**

Audio-visual alcohol content, including branding, is prevalent in UK television, and is therefore a potential driver of alcohol use in young people. These findings are virtually unchanged from our earlier analysis of programme content from 2010.

## Introduction

It is estimated that per capita alcohol consumption of pure alcohol in the those over the age of 15 years in the UK is 12.3 l,^1^ the eighth highest rate in Europe.^2^ Alcohol use was responsible for at least 6813 deaths in the UK in 2015,^3^ and cost the NHS £3.5 billion in 2013–14.^4^ It is estimated that 1.6 million people in the UK have some form of alcohol dependence.^4^ Preventing morbidity and mortality from alcohol consumption is therefore a public health priority.

Since initiation of alcohol use at a young age is a strong risk factor for dependence in later life,^5^ it is important to identify avoidable causes of alcohol consumption in young people in the UK. There is strong evidence that exposure to advertising or other alcohol imagery in the media increases subsequent use in adolescents.^6–24^ An estimated 28 million British homes have at least one television^25^ and in 2015 average viewing was 3 h, 47 min per person per day.^26^ The Office of Communications (OfCom) Broadcasting Code^27^ (Section 1.10), protects under-18s by restricting depictions of alcohol use in programmes made for children and preventing the glamorization of alcohol use in programmes broadcast before the 9 pm watershed,^28^ or in programmes likely to be widely seen, heard or accessed by under-18s without editorial justification. However, 13% of 4–15-year olds view programmes after the 9 pm watershed^29^ when programmes unsuitable for children are allowed to be broadcast.^28^ There are currently no restrictions on when commercial alcohol advertisements can be scheduled,^30^ and whilst alcohol advertisements largely adhere to existing regulations,^31^ this does not adequately protect against content that promotes irresponsible and unhealthy consumption,^31^ particularly on adolescent drinking behaviours.^13–20^ Furthermore, an estimated 62% of 12–15-year olds have access to a TV in their bedroom, and 45% of children watch TV on devices such as tablets, allowing TV viewing without parental control.^29^ Studies in earlier decades have found that alcohol imagery appeared frequently in studies of UK television,^32–36^ and the most recent content analysis of UK broadcast TV found that 40% of programmes contained alcohol content,^36^ however, there are no contemporary data on the amount of alcohol imagery and paid-for advertising in current TV programming.

While there have been no specific regulation changes since the previous content analysis in 2010,^36^ it is important to monitor a potential risk to public health to ensure compliance with the OfCom guidelines^27^ (Section 1.10). We have therefore quantified the content of all programmes and advertisements/trailers broadcast on all five national UK free-to-air channels in 2015 to document the amount of alcohol imagery contained and to explore differences in content between channels and genres, and compared these with the findings of our 2010 study.^36^

## Methods

All programmes and advertisements/trailers broadcast on the five national UK free-to-air channels in the UK (BBC1, BBC2, ITV1, Channels 4 and 5) were recorded during the peak viewing hours of 6–10 p.m. capturing data on the 3 h before and 1 h after the Office of Communications (OfCom) 9 pm watershed. All programmes, and all advertisements and/or trailers for other programmes broadcast in these time intervals were recorded in three separate weeks (Monday–Sunday) in 2015, with a 4-week gap between each (21–27 September, 18–25 October, 16–22 November). At the time of the study these five channels were the most watched channels in the UK,^37^ and remain so today.^38^ Two of the channels, BBC1 and BBC2, are public service channels with no commercial advertising, while ITV1, Channels 4 and 5 all feature commercial advertising. All recorded footage was viewed and coded using the 1-min interval period method previously described by Lyons *et al.*^36^ The method includes recording the presence or absence of audio-visual alcohol content every 1-min and in the following categories:

*Actual use*: Use of alcohol on screen by any character
*Implied use*: Any inferred alcohol use without any actual use on screen.
*Other alcohol reference*: The presence on screen of alcohol or other related materials
*Brand appearance*: The presence of clear and unambiguous branding

Alcohol appearances were recorded if they appeared on screen in any 1-min coding period. Multiple instances of the same category in the same 1-min period were considered a single event, however, if two instances of different categories occurred this was recorded as two different events (e.g. actual alcohol use and inferred use). When the same appearance transitioned into a new 1-min period it was coded as two separate events as the appearance occurred in two, 1-min intervals. We categorized the genre of each programme based on information from Box of Broadcasts^39^ and the researcher’s discretion when genre not available. The periods between programmes containing trailers for forthcoming broadcasts on BBC1 and BBC2, and those including trailers and/or commercial advertising between or within programmes on ITV1, Channel 4 or Channel 5 were coded separately from programmes as a single category of advert/trailer content. One-third of the recorded footage was coded separately by two authors (A.B. and K.W.) to ensure accuracy and reliability in the coding method. Data were analysed descriptively with separate analyses conducted to compare content broadcast before or after the 9 pm watershed, between the five channels, and between the programme genres observed.

## Results

A total of 611 programmes and 1140 advert/trailer periods comprising 27 083 1-min intervals (22 960 from programmes and 4123 from adverts/trailers) were recorded during the peak viewing hours of 6–10 pm from Monday to Sunday in 3 separate weeks. The most frequent programme genres were News and Current Affairs, Documentaries, and Soap Operas (totalling 137, 126 and 76 programmes respectively). The genres accounting for the greatest broadcast time were Documentaries, News and Current Affairs, and Entertainment, with 5482, 3573 and 2408 min, respectively.

### Any alcohol content

**Fig. 1:**
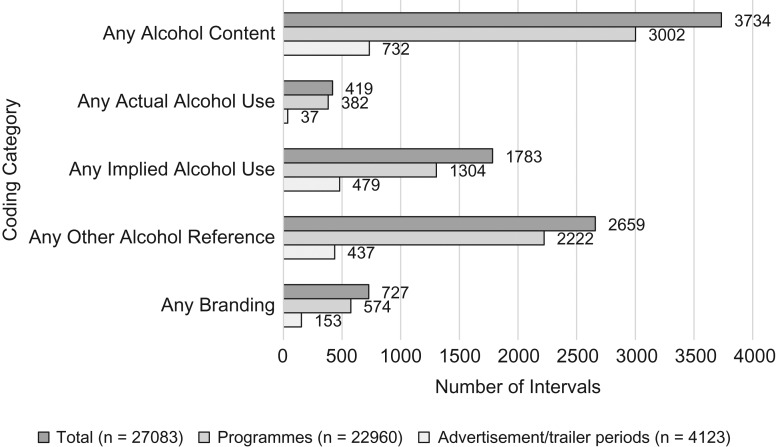
Number of 1-min intervals containing alcohol content coded by programmes or advertisements/trailers.

Any alcohol content appeared in a total of 3734 intervals (14% of the total) (Fig. 1). Of the 611 programmes broadcast, 412 (67%) contained any alcohol content, occurring in 3002 (14% of all intervals from programmes) 1-min intervals. Of advert/trailer periods, 524 (47%) contained any alcohol content, occurring in 732 (18% of all intervals from advert/trailer periods) 1-min intervals.

**Fig. 2:**
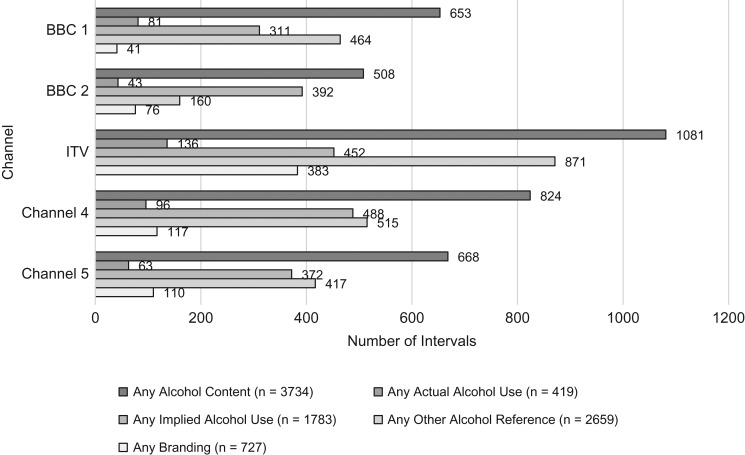
Number of intervals that contain alcohol on each channel, by coding category.

The channel broadcasting the greatest number of alcohol appearances was ITV, which accounted for 1081 intervals (29% of all alcohol intervals) and with alcohol appearances occurring in 226 programmes (62% of all broadcasts on ITV). ITV also had the most alcohol appearances in both programmes (20% of all intervals from programmes, 83% of all programmes) and advert/trailer periods (55% of all advert/trailer periods, 18% of all intervals from advert/trailer periods containing alcohol). The lowest number of alcohol appearances occurred on BBC2, with 508 intervals (14% of all alcohol intervals) across 91 broadcasts (40% of all broadcasts). BBC2 also had the lowest number of alcohol appearances in both programmes (63% of all broadcasts, 10% of all programme intervals containing alcohol) and advert/trailer periods (15% of all advert/trailer periods, 7% of all intervals from advert/trailer periods containing alcohol; see Table S1 in the online Supplementary file 1). ‘Other Alcohol References’ were the most frequent form of alcohol appearance on each channel. When compared to an earlier analysis of UK TV footage using the same methods, the results appear to be virtually unchanged, see Fig. 2.

**Fig. 3:**
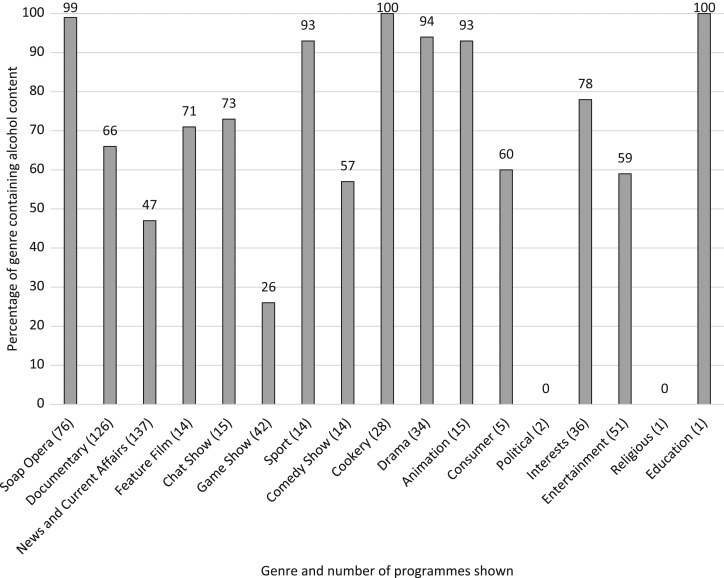
Percentage proportion of genres containing alcohol content.

The proportion of programme genres containing the highest alcohol content, defined by any alcohol content in the show, were Cookery (all cookery programmes included alcohol content), Soap Opera (99% included alcohol content) and Drama (94% included alcohol content) (Fig. 3; note that the high proportion of Education programmes containing alcohol arises from a single programme).

Of the 1120 advert/trailer periods analysed, 524 (47%) contained any alcohol content, occurring in 732 (18%) of 4123 1-min intervals. The 75% of programming in our sample broadcast before the 9 pm watershed contained 78% of the broadcast alcohol appearances in 1-min intervals.

### Actual alcohol use

Actual alcohol use occurred in 419 1-min intervals (2% of all intervals) across 147 programmes (24% of total programmes) and 37 advert/trailer periods (3% of total advert/trailer periods). The highest proportion of alcohol use involved consumption of beer or cider (177 intervals, 39% of the total intervals containing alcohol use) followed by wine or champagne (139 intervals, 31%); spirits (99 intervals, 22%); and cocktails (20 intervals, 4%). There were 16 intervals in which the alcohol type was not clearly identifiable. Alcohol use occurred in 16/20 genres and was most prominent in Drama (62% of total drama programmes), Soaps (61%) and Films (57%). The 75% of programming in our sample broadcast before the 9 pm watershed contained 77% of the broadcast alcohol appearances. Eight films (57% of all films coded) contained alcohol use, six of which were shown pre-watershed. All were classified as suitable for viewing by children by the British Board of Film Classification (BBFC) (one of them classified as ‘U’ with no age restriction) (Table S2 in the online Supplementary file 2). The majority of scenes involving alcohol use featured adults (404 instances, 96%); there were 18 instances of alcohol use by people who appeared to be under the age of 18 years.

### Implied alcohol use and other alcohol references

Implied alcohol use occurred in 1783 intervals (7% of the total intervals) and in 669 of all broadcasts (38% of all programmes/advert/trailer periods combined). Implied use typically involved holding, but not consuming, an alcoholic drink (1452 intervals; 69% of other references). Other alcohol references, which typically involved the appearance of beer pumps or glasses, occurred in 2659 1-min intervals (10% of all intervals) and in 700 programmes and advert/trailer periods broadcast (40% of all programmes/advert/trailer periods combined).

### Alcohol brands

Alcohol branding appeared in 727 1-min intervals (3% of all total intervals), across 111 programmes (18% of all programmes) and 123 advert/trailer breaks (11% of all advert/trailer periods). In total, 122 brands were identified, the most common of which was *Heineken*, which was shown in 339 intervals (46% of all intervals containing branding). Some brands were fictitious: for example, ‘The Simpsons’ programme featured *Duff* beer, *Moe et Chandon* champagne and *Absolut Krusty* vodka. Whilst real alcohol brands (including *Carling*, *Strongbow*, *Jack Daniels* and *Jim Beam*) were observed in soap operas, the majority of branding observed in soaps was fictional (*Clayton Beer* (in Emmerdale); *Bramford Beer* (in Eastenders)).

Branding within programmes pre-watershed was found in 511 intervals (3% of pre-watershed intervals) in 95 programmes (19% of the total pre-watershed programmes). The majority of branding was in the form of commercial adverts (624 1-min intervals, 86% of the total branding intervals) followed by branding on items not used in a scene (62 1-min intervals, 9% of the total branding intervals), branding on a product used in a scene (32 intervals, 4% of the total branding intervals) and 26 ‘other’ 1-min intervals (4% of the total branding intervals) which included branding such as verbal mentions.

**Fig. 4:**
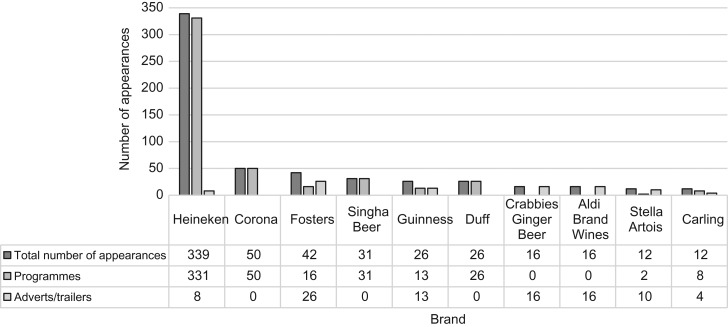
Number of appearances for the 10 most common brands by advertisement or during a programme.

The most common form of branding within programmes was sponsorship (484 intervals, 67% of all branding intervals), either of a programme (e.g. Channel 4’s Original Comedy sponsorship with *Fosters*^40^) or through branding displayed on billboards during sporting events (such as *Heineken’*s sponsorship of the 2015 Rugby World Cup^41^) followed by products used in scene (21 intervals) and products not used in scene (57 intervals). *Heineken* branding accounted for 38% of appearances (Fig. 4), which arose mostly from the 2015 Rugby World Cup sponsorship, where Heineken branding was displayed on billboards during the event, which alone generated 313 pre-watershed appearances.

In total there were 153 advertisement breaks featuring commercial advertisements containing alcohol branding, 103 of which were broadcast pre-watershed (67% of the total commercial advertisements containing alcohol branding). In total, 30 commercial alcohol adverts featuring branding also contained alcohol use. The most common commercial brand shown was *Fosters*, which occurred in 16 1-min intervals (4 pre-watershed). The majority of brand appearances occurred in programmes rather than commercial advertising (Fig. 4).

### Comparison to previous analysis

In comparison to the previous content analysis of UK broadcast television, the number of intervals containing any alcohol content increased from 2947 intervals to 3734 intervals, though largely as a result of increases in the number of intervals containing implied use or other alcohol references (see Table S3).

## Discussion

### Main findings of this study

This study demonstrates that alcohol imagery is extremely common on UK television, occurring in more than half of all programmes and almost half of all advert/trailer periods broadcast in peak viewing time, and with similar frequency both before and after the 9 pm watershed, on the most popular television channels. While alcohol branding was uncommon, over 100 brands were identified and some, such as *Heineken*, appeared very frequently. The amount of any alcohol content was slightly higher, primarily as a result of implied use or other alcohol references, relative to our earlier analysis of programming from 2010,^36^ suggesting that UK prime-time television remains a constant source of exposure to alcohol imagery for young people. The majority of brand appearances occurred in programmes rather than commercial advertising, suggesting investigations focussing on alcohol advertisements, such as those conducted by OfCom,^42^ are missing a great deal of alcohol brand content. Of the brands identified, *Heineken*, *Corona* and *Fosters* combined accounted for approximately half of all the total branding identified. However, third most common brand in total, *Fosters*, was the most common in adverts/trailers. The most common brand in total, *Heineken*, was mostly featured within actual programmes as a sponsor. Commercial adverts for alcohol were commonly aired before the 9 pm watershed.

The majority of branding was broadcast in programme content rather than in advertising, and occurred through the sponsorship of programmes, such as comedy on Channel 4 (sponsored by *Fosters*^40^), or branding featured in coverage of sports events, such as *Heineken*, which featured predominantly during the Rugby World Cup footage.^41^ Advertisements are regulated by the Advertising Standards Authority (ASA) and are expected to conform to the UK Code of British Advertising (BCAP code). According to the code, alcohol ‘may not be advertised in or adjacent to children’s programmes or programmes commissioned for, principally directed at or likely to appeal particularly to audiences below the age of 18’ (Rule 32.2). Further, advertisements must also not ‘appeal strongly to people under 18, especially by reflecting or being associated with youth culture or showing adolescent or juvenile behaviour’ (Rule 19.15.1). However, programmes popular with or watched by large numbers of young people are not necessarily made specifically for them. The ASA’s definition of advertising^30^ also does not include sponsorship of programmes or pitch side advertisements at televised sporting events. Exemption of these programmes from the above constraint has the potential to lead to significant exposure among young people during peak viewing hours, when ~4.5 million 7–14-year olds watch television.^29^

### What is already known on this topic

Initiation of alcohol use at a young age is a strong risk factor for dependence in later life^5^ and there is strong evidence that exposure to advertising or other alcohol imagery in the media increases subsequent use in adolescents.^6–24^ Studies in earlier decades have found that alcohol imagery appeared frequently in studies of UK television.^32–36^ The latest content analysis of UK broadcast TV was conducted in 2010 and found that alcohol content occurred in 40% of programmes, and was equally frequent both before and after the 9 pm watershed.^36^

### What this study adds

Alcohol content shown on TV has an effect on the uptake of alcohol use in young people.^6–24^ Our analysis shows that television remains a major source of alcohol exposure to young people in the UK and is likely to continue to be a contributor to alcohol uptake by young people,^43^ with levels of content slightly higher than observed in our earlier analysis of programme content from 2010.^36^ The OfCom 9 pm watershed, designed to protect children and young people from potentially harmful imagery,^28^ is currently not fulfilling its purpose in relation to commercial advertising and alcohol branding in programmes. Quantifying AVC content can be used to impact policy and change depictions in the media.^44–46^ Tighter scheduling rules from the ASA and OfCom, such as restricting alcohol content to after the 9 pm watershed, could prevent children and adolescents being exposed to alcohol content and advertising.

### Limitations of this study

We coded terrestrial channels since the majority of households have access to these channels and programmes aired on these channels consistently receive the highest viewing share in the UK.^37^ Given the large viewing audience of these channels, and the fact that the majority of viewing by 4–15-year olds in the UK occurs in early morning or early evening (7–9 pm),^29^ we assumed that children and young adults will have access to, and be viewing, the five main channels. However, we acknowledge that it is equally likely that children will have access to and view other channels. We chose to code programmes broadcast between 6 and 10 pm since programmes shown in this time period are the most viewed throughout the week. While the current study only explored UK broadcast TV, the literature suggests links between exposure to alcohol AVC and subsequent use in studies conducted around the world.^6–24^

## Supplementary Material

fdy142_Table_S1

fdy142_Table_S2

fdy142_Table_S3
