# Supplementary material for: A content analysis of alcohol content in UK television
Source: J Public Health (Oxf). 2018 Oct 14;41(3):462–9. doi: 10.1093/pubmed/fdy142 (PMC6785681; doi:10.1093/pubmed/fdy142)
Supplement: fdy142_Table_S1 [file fdy142_table_s1.docx]

| **Channel** | **Proportion of total intervals containing alcohol references** | | **Proportion of programme intervals containing alcohol references** | | **Proportion of advert/trailer intervals containing alcohol references** | |
| --- | --- | --- | --- | --- | --- | --- |
|  | **Intervals**  **Number of intervals/Total amount of intervals (%)** | **Programmes (%)**  **Number of intervals/Total amount of intervals (%)** | **Intervals (%)**  **Number of intervals/Total amount of intervals (%)** | **Programmes (%)**  **Number of intervals/Total amount of intervals (%)** | **Intervals (%)**  **Number of intervals/Total amount of intervals (%)** | **Programmes (%)**  **Number of intervals/Total amount of intervals (%)** |
| BBC1 | 653/5214 (13%) | 109/226  (48%) | 637/4982 (13%) | 87/124  (70%) | 22/232 (11%) | 22/101  (21%) |
| BBC2 | 508/5167 (10%) | 91/228  (40%) | 491/4879 (10%) | 74/117  (63%) | 16/228 (7%) | 16/110  (15%) |
| ITV | 1081/5456 (20%) | 226/362  (62%) | 888/4335 (20%) | 91/109  (83%) | 193/1066 (18%) | 135/224  (55%) |
| Channel 4 | 824/5547 (15%) | 252/406  (62%) | 578/4338 (13%) | 90/108  (83%) | 246/1209 (20%) | 162/298  (54%) |
| Channel 5 | 668/5699 12% | 267/518  (52%) | 417/4311 (10%) | 78/151  (52%) | 251/1388 (18%) | 189/367  (51%) |

**Table S1:** Proportion of intervals containing alcohol content by channel
