# Supplementary material for: A content analysis of alcohol content in UK television
Source: J Public Health (Oxf). 2018 Oct 14;41(3):462–9. doi: 10.1093/pubmed/fdy142 (PMC6785681; doi:10.1093/pubmed/fdy142)
Supplement: fdy142_Table_S2 [file fdy142_table_s2.docx]

| **Film Title** | **Number of intervals containing alcohol content** | **BBFC age classification** | **Channel** | **Watershed** |
| --- | --- | --- | --- | --- |
| Midnight’s Children | 13 | 12 | BBC 2 | Pre-watershed |
| Skyfall | 11 | 12 | ITV | Post-watershed |
| R.E.D | 3 | 12 | ITV | Post-watershed |
| Snow White and the Huntsman | 17 | 12 | Channel 4 | Pre-watershed |
| Jackie Chan’s The Forbidden Kingdom | 8 | 12 | Channel 5 | Pre-watershed |
| Peter Pan | 4 | U | Channel 5 | Pre-watershed |
| Rush Hour | 8 | 15 | Channel 5 | Pre-watershed |
| Just Go With It | 15 | 12 | Channel 5 | Post-watershed |

**Table S2:** Age rating, channel and time aired of the films broadcast.
