# Supplementary material for: A content analysis of alcohol content in UK television
Source: J Public Health (Oxf). 2018 Oct 14;41(3):462–9. doi: 10.1093/pubmed/fdy142 (PMC6785681; doi:10.1093/pubmed/fdy142)
Supplement: fdy142_Table_S3 [file fdy142_table_s3.docx]

**Table S3: Comparison of the number of one-minute intervals containing alcohol content in 2010 and 2015**

|  | Number of one-minute intervals from the 2010 analysis | Number of one-minute intervals from the 2015 analysis |
| --- | --- | --- |
| Any Alcohol Content | 2947 | 3734 |
| Actual Alcohol Use | 478 | 419 |
| Implied Alcohol Use | 1542 | 1783 |
| Other Alcohol Reference | 2209 | 2659 |
| Alcohol Branding | 1051 | 727 |
